# Considerations on the State of Medicine in France, from the Time of the Revolution to the Present Period

**Published:** 1819-12

**Authors:** J. B. Regnault

**Affiliations:** Consulting Physician to the King, &c. &c.


FOR THE LONDON MEDICAL AND PHYSICAL JOURNAL.

### *Considerations on the State of Medicine in France, from the Time of the Revolution to the present Period.* By J. B. REGNAULT, M.D. Consulting Physician to the King, &c. &c.

[*Concluded from page* 368.]

THE inclination to method and classification that the taste for natural history had rendered prevalent, and which followed so naturally from the labours of the philosophers of the eighteenth century, excited a desire for a classification of diseases more regular than those of SAUVAGES and CULLEN: this has been realized by the publication of a work* regarded with enthusiasm on its appearance, afterwards considered as a medical code almost sacred, but at length attacked with violence even by those who were originally disciples of the author. This work has all the faults, with all the advantages, attached to a work of this kind; but it is incomparably better than all those of the same species which had been published since the first essay of PLATER. We cannot here avoid expressing our regret at the manner with which some physicians have endeavoured to deprive an author, justly celebrated, of the reputation which he had acquired by his honourable labours, but which could not prevent his falling into some few errors.

Fevers, formerly distributed into many classes, have appeared, for some years past, to be at length reduced to fixed and determined species. Practitioners, however, but rarely find at the bedside of the patient those pictures of simple disease so well designed in books of nosography, where the authors appear to have forgotten the numerous complications which morbid affections acquire. The discredit into which the best and most

---

* The *Nosographie Philosophique,* of M. PINEL.

recent writers on pyretology has begun to fall, proves that the hope ever to find one which may be free from censure was really vain.

To laborious efforts at classification have succeeded others, more fortunate, to demonstrate the uncertainty of all distinctions of fevers, solely founded on the consideration of their symptoms. The investigation of the seat of diseases, which the ancients always earnestly advised, and which physicians of all ages have pursued with ardour, but too often in an erroneous manner, has been recently adopted with particular care by some accurate observers.

Pathological anatomy has been enriched by the researches of several of the most distinguished of the disciples of the new school. Of these some are now dead;* and the true friends of medical science deplore their loss; some of those who have yet survived have merited, by their persevering efforts to extend the boundaries of medicine, to take their seat amongst their masters, and to the school in which they were formed they have given a new lustre.

We do mean to say, that, until the labours of Bayle and his contemporaries, physicians had neglected searching for the seat of diseases; but we must acknowledge, that in fevers this enquiry had been neglected, or made with but little perseverance, and even with some degree of prejudice.

The history of the phlegmasiæ, on the contrary, was soon modified by the determined progress of pathological anatomy: though the distinction of the tissues, which the imagination alone can isolate, was too much insisted on, still inflammation affecting analogous organs were at least arranged together, and the exposition of their symptoms thus rendered more lucid, at the same time that their diagnosis was much facilitated.

Although we possess several good treatises on Hæmorrhages, these are perhaps the affections the history of which has been the least perfected during the last thirty years. Hitherto we have too much neglected to consider the relations between hæmorrhages, properly speaking, and sanguineous effusions into the tissue, or in the cavities, of organs not having a natural issue. This subject offers a vast scene for new researches to observers, which would not fail to furnish much useful knowledge, if we may judge from those of the same kind which have been already made.

The general history of nervous diseases has hardly been much advanced of late years: that of apoplexy is more complete in an anatomical respect, though the diagnosis of it has not been much improved. But there is one class of the neuroses, the

---

* Bayle, Marandel, Le Gallois, &c.

theory and treatment of which has received the most useful modifications. A Frenchman* has first carried the light of philosophy and the deep views of a superior mind into the history, previously hardly even sketched, of mental alienations. The plan of his treatise, become a classic work throughout all Europe, is marked in the records of our art amongst the immortal writings of great and original observers of all ages.

Some organic diseases have been studied with more care, with respect to the alterations they occasion in the tissues of the animal economy. The important works published on the diseases of the liver, the pleura, the stomach, the intestines, and the peritoneum, are well known. These different productions assure to the authors† the glory of having contributed to the progress of the healing art. The history of the diseases of the heart and the large blood-vessels, has been enriched by the researches of the able successor of Desbois of Rochefort,‡ in his clinical instruction.

An excellent treatise on the diseases of the skin,§ remarkable by descriptions as correct as they are animated, and by the manner of the author's style, has not however made us forget, although it has worthily replaced, the work of Lorry.

The knowledge of the signs of diseases, the object of constant solicitude to the ancients, has hardly been improved by the moderns, in all that relates to prognosis. Some enlightened practitioners‖ have collected and applied to the pathology of the present period all that has been acquired on this subject; and they have added many remarks of their own, which show as much correctness of judgment as talent for accurate observation.

Surgery has been enriched by several new operatory proceedings: the diseases which come within its domain have been better studied, and the curative methods have been directed by the precepts of physiology. Many treatises¶ on this branch of the healing art have appeared; and each of them merits the reputation it has obtained for the author.

Botany has received a new direction by the distribution of plants into natural families. This ingenious method, which collates the vegetables, the action of which on the animal economy is nearly similar, must be more agreeable to physicians, because it would place botany in harmony with therapeutics, if it did not also in some instances unite plants, of which the medical qualities are totally different from each other; and of which some are even more deleterious than useful. We must depend on time, and the labours of our indefatigable phytolo-

* M. Pinel.

† Bayle, Portal, Baumes, Broussais, and Laennec.

‡ M. Corvisart. § Of M. Alibert. ‖ MM. Double and Landré Beauvais.

¶ By MM. Boyer, Delpech, and Richerand.

gists, for the perfectionment of this system, so seducing in theory.

The discoveries in chemistry during the last thirty years have been almost infinite; and it every day makes a new progress, to be followed by yet new ones. Will medicine derive from it any advantage? We may doubt of it, when we consider that the knowledge of affinities has not elucidated in any way the mechanism of the vital functions, nor thrown any light on the diagnosis and the causes of diseases.

An author,* very recently, well known by his superior talents, has shown the slight foundation of the chemical explanations proposed respecting the vital phenomena.

Chemistry has taught us to understand better the qualities of the simple and compounded minerals, the immediate principles of vegetables employed as medicines; it has traced laws that are certain, for the mixture of different substances without their decomposition: yet, we must say, the art of prescribing proper formulæ becomes daily less familiar to the pupils of our faculties.

Medical jurisprudence has been cultivated in France with more care than formerly: the example of the Germans has not been lost to us. Men of eminent merit† have made it the especial object of their researches. There is one point in this science in which the French have shown themselves even superior to the Germans: this is toxicology. The action of poisonous substances on the human body has been studied according to the principles of physiology. Experiments have been made on animals: the progress of chemistry has furnished certain means for the detection of poisonous substances in the tissues of our organs, or in the fluids administered by criminal hands.

It has here been made apparent, that French physicians, before the Revolution, confined themselves, generally, to the methodic use of the curative measures of which experience had demonstrated the efficacy. This course, the only good one, the only one that is advantageous, has been viciously modified by the introduction of the system of Brown into France; although this assertion, which had been already made by one of our most distinguished physicians, has been protested against: but it is a truth so completely demonstrated, in the opinion of all judicious men, that it will soon become of but trifling importance.

The Scotch reformer, in reducing the whole pathological list to two series, held out, to those physicians who were seduced by the simplicity of such an arrangement, the attractive hope of imparting the same simplicity to therapeutics: there

* M. Coütanceau.

† Beloc, Mahon, Chaussier, Foderé, Orfila, &c.

were very nearly being only two diseases and two remedies. They went further: they persisted in seeing, in almost all cases, no other affection than a want of energy. Bleeding was proscribed. Every experienced physician who hazarded the prescription of it, was designated by the public as an imprudent practitioner, imbued with superannuated errors. They went so far as to assert, that this operation was but rarely proper in inflammations of the chest: cerebral congestions even were treated with cordials.

It would be wrong to conclude, from what we have just said, that medicine presents a fearful versatility, and that the reveries of a rash innovator are sufficient to make physicians abandon the paths of experience, and lead the most grave and sedate of them into the mazes of error.

When Brown attacked the bases of a science founded on the observations of twenty ages, he, without doubt, drew to his side many minds more ardent than well-disciplined, more brilliant than profound, which had not been enlightened by experience: but his ambitious efforts, and the language of his adepts, could effect nothing on the minds of the greater number of physicians, who had acquired from the ancient school a salutary defiance for all that bears the character of novelty, for all that is totally different from what the experience of ages has proved to be good and useful, and for all that has exclusive pretensions. The voices of these prudent practitioners were certainly often stifled ; but, we frankly assert, one man has presented himself who has ventured to take on himself the cause of their antagonists.* His glory would have been without blemish, if he had not in some measure sullied so fine a cause, by some instances of want of propriety in the manner in which he proceeded in it: this, time will at length dispel. This intelligent physician has shown, in the most forcible manner, the importance of investigating the seat of diseases, the utility of antiphlogistic measures in a great number of cases where they were proscribed by views purely speculative: he has made it appear that, in fevers, inflammation plays a great part; and that it is necessary to attend to this in the choice of the curative measures: he has shown the way in which many species of chronic inflammation manifest themselves, and the modifications required in the treatment of diseases, by the nature of the tissue in which they are seated. Such are his incontestible titles to celebrity ; but nothing can authorize us to see, in almost all cases, merely irritation, and the necessity of abstracting a large quantity of blood. We think that every exclusive notion, every method too much generalized, presents something incompatible

* M. Broussais.

with truth; and, if we have blamed Brown for having seen only debility, we should also blame those who see every-where nothing but exaltation of vital action. However, this is less addressed to the intelligent physician to whom I have alluded, than to many of his disciples, whose indiscreet zeal requires to be moderated, that it may be retained within the boundaries of utility and truth.

The most important of the modifications introduced into medicine during the last thirty years, is the new mode of instruction. We do not think, however, that it is now as good as it might become; but it cannot be denied, that it is infinitely superior to that in use before the Revolution. At that period, as we have already shown, good physicians formed themselves, or at least with the aid of some few men influenced by the aid of their own genius, not by the power of public instruction. Now, all pupils receive solid instruction, which places them in the true route of observation and experience.

Natural history, chemistry, botany, and zoology, are at the present time taught with the greatest *eclat* by some men of science,* whose labours have contributed to the glory of our nation.

Numerous courses of anatomy much facilitate the study of this science. Every pupil investigates with the scalpel the structure of the different organs; he examines them himself, isolates them from others, and thus impresses on his memory the picture of their formation.

Physiology may enumerate several professors† who have contributed much to the improvement of this science, and many young physicians‡ who, in their private courses of Lectures, announce a maturity of talent at an early age of life.

Hygiene, formerly so neglected, at least at Paris, has lately been taught in a brilliant manner by a professor,§ justly celebrated, whose silence in other respects now excites so much regret, since his work, so long expected with eagerness, has not yet appeared.

Clinical courses on medicine and surgery draw after the steps of experienced and judicious practitioners‖ a crowd of pupils. The impulse given by Desbois of Rochefort, and Desault, is still felt, because it has been favoured and kept up by the zeal and great talents of their successors. However, the teaching of practical medicine is not yet what it should be; and it is in this point especially, that our institutions should hope to be perfected in a manner so ardently desired by all men of judgment.

---

* Biot, Gay-Lussac, Thenard, Richard, De Jussieu, Des Fontaines, and Blainville.

† Chaussier, Richeraud, Beclard, &c.

‡ Adelon, Rullier, and Richeraud. § Hallé.

‖ Petit, Recamier, Leroux, Fouquier, Broussais, Boyer, Dubois, Dupuytren, &c.

Why should one of the most interesting parts of medical science be still so neglected. The natural history of medicine is very well known; but whilst the operatory measures, the cases in which they should be resorted to and where they should be abstained from, and the different other surgical means, are exposed with so much sagacity by the most eminent surgeons of the ancient and new school of Paris, a course, in which the true principles of medical therapeutics, developed with that superiority of views that marks an enlightened practitioner, yet remains to be desired. This blank in instruction depends, in a great degree, on the extent to which scepticism in this respect has been carried. The new works we have on the materia medica and therapeutics, seem intended to destroy our confidence in the agency of medical substances, rather than to show those which should be resorted to, and the rules for their administration.

It would be wrong, to make our knowledge in this respect consist in all the trash which authors have proposed; but, without adding in a ridiculous manner recipe to recipe, and without attributing to remedies all the properties which have been pompously given to them by credulous and enthusiastic writers, could we not, in the present day, collect a complete body of doctrine of all that observation and close induction has taught respecting the medicinal qualities of the agents of which the description constitutes the materia medica, and on the art of applying them to the human body, according to the indications and the rules of therapeutics.

For many years the study of the diagnosis of disease has absorbed the general attention: men have occupied themselves with much care in acquiring a knowledge of the name which may be given to them, and have too much neglected the principles on which reposes the knowledge that teaches how they may be cured. These principles are well known by the practitioners who transmit them to their pupils in their lectures, and by their example; but a regular system of instruction is yet wanting on this the most important part of medicine, since it is this which properly constitutes the knowledge of the physician, and which is the summary of all the branches of medical science, which in a manner only serve as introductions to it. When we shall have nothing to desire in this respect, at least as far as human knowledge will permit, then alone shall we arrive at a new epoch in the history of medicine. This epoch will be characterized by the happy alliance of practice with theory. Every thing, however, seems to indicate that the moment is arriving, when this salutary object, laboured for during so long a series of years, will be effected.

*Paris; June,* 1819.

